# Plasmon-Induced Water Splitting on Ag-Alloyed Pt Single-Atom Catalysts

**DOI:** 10.3389/fchem.2021.742794

**Published:** 2021-10-25

**Authors:** Yimin Zhang, Daqiang Chen, Weite Meng, Shunfang Li, Sheng Meng

**Affiliations:** ^1^ Key Laboratory of Material Physics, Ministry of Education, School of Physics and Microelectronics, Zhengzhou University, Zhengzhou, China; ^2^ Beijing National Laboratory for Condensed Matter Physics and Institute of Physics, Chinese Academy of Sciences, Beijing, China; ^3^ School of Chemical Engineering, Anhui University of Science and Technology, Huainan, China; ^4^ School of Physical Sciences, University of Chinese Academy of Sciences, Beijing, China

**Keywords:** photocatalytic water splitting, localized surface plasmon, single-atom catalyst, charge transfer, time-dependent density functional theory

## Abstract

A promising route to realize solar-to-chemical energy conversion resorts to water splitting using plasmon photocatalysis. However, the ultrafast carrier dynamics and underlying mechanism in such processes has seldom been investigated, especially when the single-atom catalyst is introduced. Here, from the perspective of quantum dynamics at the atomic length scale and femtosecond time scale, we probe the carrier and structural dynamics of plasmon-assisted water splitting on an Ag-alloyed Pt single-atom catalyst, represented by the Ag_19_Pt nanocluster. The substitution of an Ag atom by the Pt atom at the tip of the tetrahedron Ag_20_ enhances the interaction between water and the nanoparticle. The excitation of localized surface plasmons in the Ag_19_Pt cluster strengthens the charge separation and electron transfer upon illumination. These facts cooperatively turn on more than one charge transfer channels and give rise to enhanced charge transfer from the metal nanoparticle to the water molecule, resulting in rapid plasmon-induced water splitting. These results provide atomistic insights and guidelines for the design of efficient single-atom photocatalysts for plasmon-assisted water splitting.

## Introduction

Photoinduced water splitting is a feasible way to mitigate the energy crisis and the associated environmental issues (Lewis, 2007). Given the high atom utilization efficiency, unique electronic structure, precisely identified active site, and excellent catalytic activity and selectivity, single-atom catalysts (SACs) have emerged as a new frontier in heterogeneous catalysis including photocatalytic water splitting in recent years (Qiao et al., 2011; Lang et al., 2020; Zhuo et al., 2020). As reported by Yang et al., compared to atomically dispersed Pd and Rh, single Pt sites anchored on TiO_2_ exhibit excellent efficiency, high stability, and photo-corrosion resistance for solar-driven water splitting (Xing et al., 2014). Afterward, a series of single-atom catalysts on different substrates had sparked tremendous attention in photoinduced water splitting (Sui et al., 2017; Wu et al., 2018; Hejazi et al., 2020). For instance, Schmuki et al. found that the rate of water splitting on the Pt site of the thin TiO_2_ layer was enhanced 150 times higher than that on Pt nanoparticles (Hejazi et al., 2020). However, these processes face great challenges in suppressing the recombination of photogenerated carriers and further enhancing the efficiency of charge transfer and chemical reactions. One way to address this weakness is to combine the advantages of SACs and plasmonic excitations in metal clusters to strengthen the light-matter interactions, thanks to the superior optical absorption and extended lifetime of excited carriers of the latter (Nie and Emory, 1997; Xu et al., 1999; Prodan et al., 2003).

Plasmonic metal clusters such as Au, Ag, Cu, and Al can concentrate and channel the energy of solar light into the absorbates after plasmon excitation, which have been prevalently utilized in chemical and solar energy conversion, especially in plasmon-driven photocatalysis including O_2_ dissociation (Christopher et al., 2011; Christopher et al., 2012; Seemala et al., 2019) and H_2_O splitting (Liu et al., 2011; Primo et al., 2011; Thimsen et al., 2011; Warren and Thimsen, 2012; Qian et al., 2014; Sigle et al., 2015; Yan et al., 2016; Yan et al., 2018). However, plasmon-induced photocatalysis using SACs has seldom been investigated, especially its underlying carrier dynamics and reaction mechanism. Mark et al. reported a theoretical study about the plasmon-mediated N_2_ dissociation on a single-atom Fe-functionalized Au cluster (Martirez and Carter, 2017). It was revealed that the strong localized surface plasmon of Au and the active Fe site worked together to lower the dissociation barrier after the consecutive resonance energy transfer. However, the carrier dynamics and underlying charge transfer mechanism was absent. Zhou et al. quantitatively explored the hot carriers and thermal contributions in a plasmon-assisted ammonia photolysis using atomically dispersed Ru on Cu nanoparticles under light irradiation (Zhou et al., 2018). However, the detailed role of the Ru site in the reaction process remains elusive and needs to be further investigated.

Here, we investigate plasmon-induced water splitting on the Ag cluster doped by a single Pt atom at the single-molecule level using real-time time-dependent density functional theory (rt-TDDFT). Through the carrier and structural dynamics analysis, we find that the introduction of single Pt atom improves light absorption and electronic level alignment as given by the strong light-matter interactions. Specially, it opens up different charge transfer channels to magnify the charge transfer rates to water molecules, enabling high-efficiency water splitting. The aforementioned findings offer new prospects for solar water splitting and for the design of optimal photocatalysts with high efficiency.

## Results and Discussion

**FIGURE 1:**
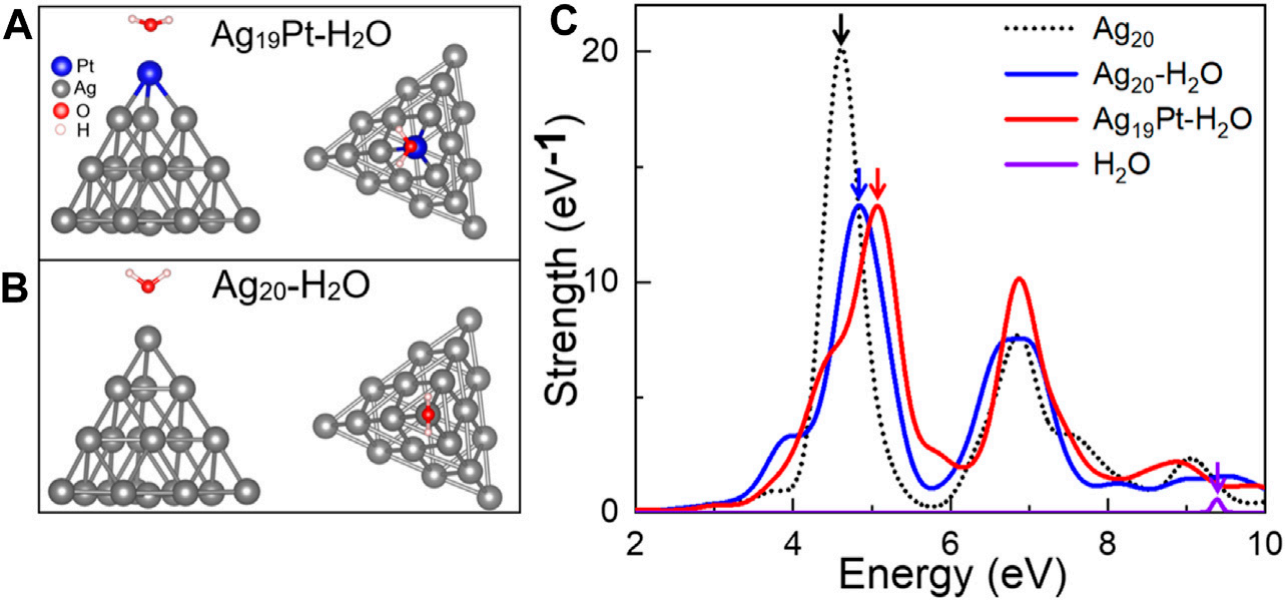
Atomic configuration and absorption spectra. The top and side views of **(A)** Ag_19_Pt–H_2_O and **(B)** Ag_20_–H_2_O after geometry optimization. **(C)** Absorption spectra of Ag_20_, Ag_20_–H_2_O, Ag_19_Pt–H_2_O, and H_2_O, respectively. The colored arrows denote the position of the corresponding absorption peak.

**Atomic Configuration and Absorption Spectrum**. Here, the tetrahedral Ag_20_ cluster is used as a model system, while larger clusters such as Ag_55_ are also tested. The reasons for choosing Ag_20_ are as follows: first, the tetrahedral Ag_20_ nanoparticle is structurally stable among many other clusters (Wang et al., 2003). Second, it has been widely used as a good model system for investigating the interactions between small molecules and metal clusters under illumination (Zhao et al., 2006a; Zhao et al., 2006b). Third, the tetrahedral Au_20_ clusters have already been obtained experimentally on ultrathin NaCl films (Li et al., 2020), which gives guidance for the synthesis of tetrahedral Ag_20_ on the supported substrates. After the relaxation of the adsorption geometry of the representative high-symmetry configurations, we selected the most stable atomic structures for Ag_19_Pt–H_2_O and Ag_20_–H_2_O, as shown in Figures 1A,B. Here, compared to the initial distance of 3 Å, the distance of Pt–O is 2.11 Å, less than that for Ag–O (2.24 Å), implying that there exists a stronger interaction between the Ag_19_Pt and H_2_O molecule than Ag_20_. In addition, the ∠H–O–H bond angle in water is 107.6° and 108.3° for Ag_19_Pt-H_2_O, Ag_20_-H_2_O, respectively, compared to the value of 104.5° in intact water molecules, suggesting that the water molecule is activated after adsorption.

The absorption spectra of Ag_19_Pt–H_2_O, Ag_20_–H_2_O, Ag_20_, and a freestanding H_2_O molecule have been calculated and are shown in Figure 1C. The absorption peak of freestanding H_2_O is located at >8 eV, corresponding to the transition from the highest occupied molecular orbitals (HOMO) and the lowest unoccupied molecular orbitals (LUMO) of water. Compared to the highest absorption peak located at 4.62 eV for Ag_20_, Ag_19_Pt–H_2_O and Ag_20_–H_2_O complexes both display a blue shift where the major absorption peak moves to 5.07 and 4.83 eV, respectively. This means that the Ag_19_Pt cluster has a stronger electronic coupling between the metal cluster and H_2_O molecule than the Ag_20_ cluster, which can also be confirmed by the photo absorption spectra calculated for Ag_55_Pt–H_2_O and Ag_55_–H_2_O, as shown in Supplementary Figure S1.

**FIGURE 2:**
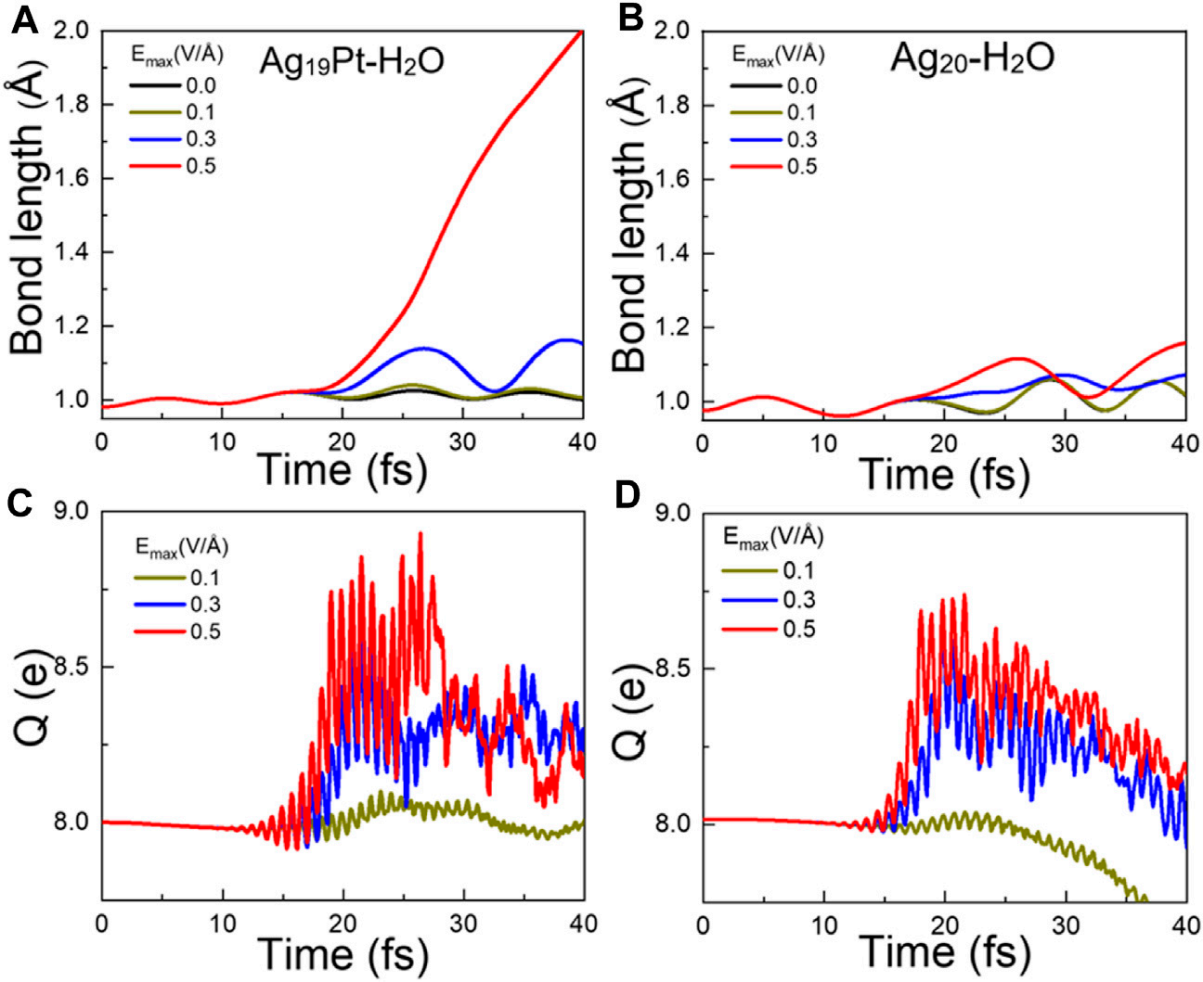
Ultrafast dynamic responses after photoexcitation. Time evolved bond length of O–H for **(A)** Ag_19_Pt–H_2_O and **(B)** Ag_20_–H_2_O under different field strengths E_max_. Time-evolved total charge (Q) located on H_2_O for **(C)** Ag_19_Pt–H_2_O and **(D)** Ag_20_–H_2_O under different field strengths.

**Ultrafast Molecular Dynamics After Photoexcitation**. To investigate the photoinduced response, we calculate the time-dependent changes in the bond length of O–H for the two complexes mentioned earlier upon laser illumination under different maximum field strength E_max_, as shown in Figures 2A,B. The couplings between atomic and electronic motions are governed by the Ehrenfest approximation (Alonso et al., 2008). Given that the equilibrium O–H bond length of gaseous H_2_O is 0.98 Å, we assume that the interaction between the OH and H atom can be considered negligible when the H–OH distance is >2.0 Å. So, for simplicity, we take the O–H bond length of 2.0 Å as the criterion for determining the breaking of the O–H bond. Obviously, one finds that without laser illumination, the O–H bond length exhibits a stable oscillation around 0.98 Å and the bond does not break, indicating that water splitting does not occur directly after adsorption on the metal clusters. Upon illumination, the O–H bond length increases with E_max_ for the two systems. In particular, the O–H bond breaks at 40 fs with E_max_ = 0.5 V/Å for Ag_19_Pt–H_2_O, while it does not break but only shows an elongation of 0.2 Å for Ag_20_–H_2_O under the same field strength. These results suggest that photocatalytic water splitting can be attributed to the cooperation of the photoexcitation and the introduction of the Pt atom.

In order to uncover the underlying mechanism of water splitting, we first compare the time-evolved total charge Q located on the H_2_O molecule under different field strengths for the two complexes, as shown in Figures 2C,D. For the two systems under irradiation, Q rises quickly when the field strength increases and then gradually decays with an oscillation to a value around 8 *e*. Here, 8 *e* corresponds to the initial total charge of a freestanding H_2_O molecule, that is, there exists a charge transfer ΔQ (ΔQ = Q − 8) from the metal cluster to the H_2_O molecule if Q > 8. In particular, compared to the charge transfer ΔQ = 0.75 *e* to the H_2_O molecule for Ag_20_–H_2_O at E_max_ = 0.5 V/Å, a charge transfer ΔQ = 0.92 *e* takes place for the Ag_19_Pt–H_2_O cluster under the same field strength, implying that the introduction of the Pt atom enhances the amount of charges transferred to water.

**FIGURE 3:**
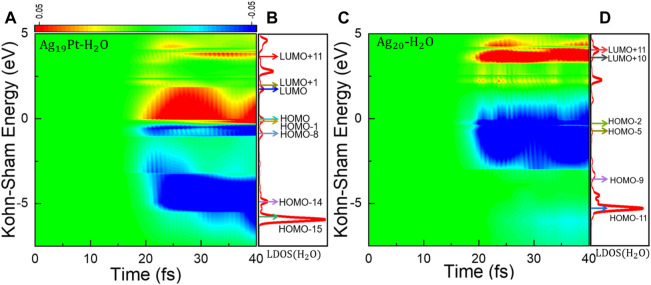
Distribution of Kohn–Sham energy levels and changes in occupation. **(A)** Time-dependent changes in the occupation of the KS states and **(B)** projected local density of states (LDOS) on the H_2_O species at *t* = 0 fs for Ag_19_Pt–H_2_O with a field strength of 0.1 V/Å. The eight arrows from the bottom to top denote the energy levels of the eight KS states, that is, HOMO-15, HOMO-14, HOMO-8, HOMO-1, HOMO, LUMO, LUMO+1, and LUMO+11, respectively. **(C)** Time-dependent changes in the occupation of the KS states and **(D)** local projected density of states (LDOS) on the H_2_O species at *t* = 0 fs for Ag_20_–H_2_O under the same condition. The six arrows from the bottom to top denote the energy levels of the six KS states, that is, HOMO-11, HOMO-9, HOMO-5, HOMO-2, LUMO+10, and LUMO+11, respectively.

**Time-Evolved Kohn–Sham States**. To further explore the difference in charge transfer for the two systems, the time-evolved occupation of Kohn–Sham (KS) states and the corresponding projected local density of states (LDOS) under a field strength of 0.1 V/Å are calculated, as shown in Figure 3. Here, the occupation of KS states is calculated by projecting the time-dependent KS state onto KS orbitals at time *t* = 0. At first, there exists an obvious oscillation in the electronic occupation for the KS states near the Fermi level for the two systems, as shown in Figures 3A,C, indicating charge density oscillations around the metal cluster surface (Townsend and Bryant, 2011). Then, the electrons at the deep energy region can be photoexcited to high-energy levels, implying the rapid plasmon decay into hot electron–hole pairs following photoexcitation (Townsend and Bryant, 2014; Ma et al., 2015). It can be seen that the energy region of excitation almost extends all over the range of 0–5 eV for the Ag_19_Pt–H_2_O cluster (Figure 3A), while it mainly locates at 3.5–5 eV for Ag_20_–H_2_O (Figure 3C), further confirming that more charge transfer to the H_2_O molecule is favored in the Ag_19_Pt–H_2_O system. In order to explain the difference in charge density oscillations for the two systems, we calculated the charge density at t = 40 fs under different field strengths, as shown in Supplementary Figure S2, indicating that the introduction of a single Pt atom alters the distribution of charge oscillations within and around the metal surface. For the sake of analysis, a weak laser pulse with a field strength of 0.1 V/Å is used. Similar results are observed under stronger laser pulses with the relative contribution of different excitation channels varying, such as the case of E_max_ = 0.5 V/Å, as shown in Supplementary Figure S3.

From the LDOS shown in Figures 3B,D, we can find that the introduction of the Pt atom displays a metallic character for the Ag_19_Pt–H_2_O cluster, while a semiconducting behavior is identified for Ag_20_–H_2_O, suggesting a qualitative change in the electronic structure. Furthermore, the LDOS located on H_2_O species is very diffusive, suggesting strong electronic couplings between water and the nanoparticle in both cases.

**FIGURE 4:**
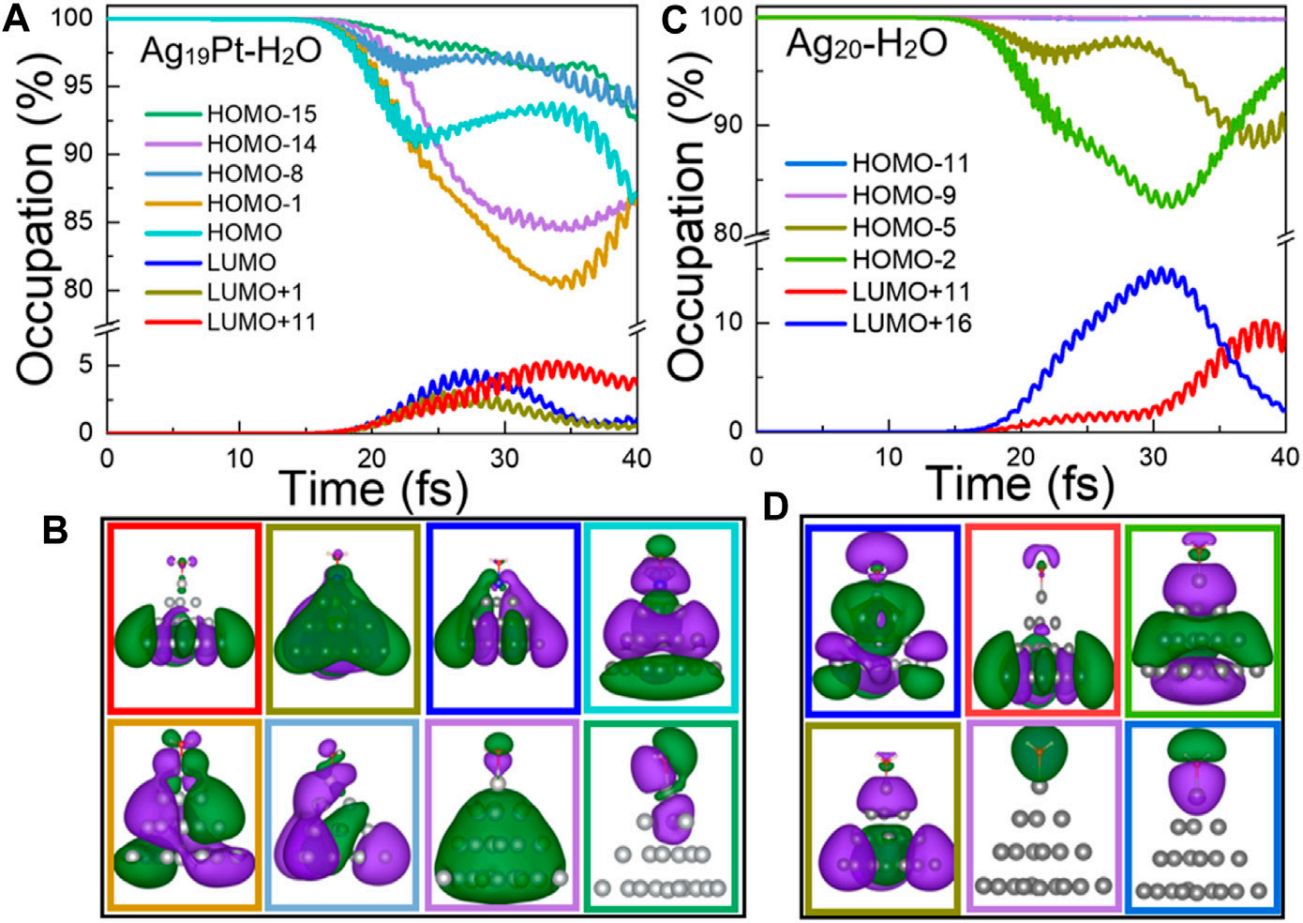
Time-evolved occupation of Kohn–Sham states and wave function plots. **(A)** Time-evolved occupation and **(B)** the corresponding wave functions of the eight states shown in different colors for Ag_19_Pt–H_2_O under a field strength of 0.1 V/Å. **(C)** Time-evolved occupation and **(D)** the corresponding wave functions of the six states shown in different colors for Ag_20_–H_2_O under the same condition. Here, the isosurface value for the wave functions is 0.02 Å^−3^.

**Time-Dependent Occupation of Kohn–Sham States**. To offer a direct description of ultrafast carrier dynamics, the time evolution of the occupation of Kohn–Sham states are shown in Figure 4. In Figure 4A, the change of occupation for the HOMO-14 and HOMO-15 (corresponding to the HOMO level of the H_2_O species as shown in Figure 4B) is 15% and 7.5%, respectively, which means that the intramolecular charge transfer exists for Ag_19_Pt–H_2_O. In contrast, the change of occupation for HOMO-9 and HOMO-11 (corresponding to the HOMO level of water as shown in Figure 4D) is 0, ruling out the intramolecular charge transfer channels for the Ag_20_–H_2_O case. In other words, the introduction of the Pt atom opens up additional charge transfer channels to facilitate the plasmon-induced chemical reaction, that is, an intramolecular charge transfer in the case of Ag_19_Pt–H_2_O. Moreover, there is a contrary variation trend between LUMO, LUMO+1, LUMO+11 and HOMO, HOMO-1, and HOMO-8 for Ag_19_Pt–H_2_O (Figure 4A), implying a charge transfer among these orbitals, so does the orbitals between LUMO+11, LUMO+16 and HOMO-2, HOMO-5 for the Ag_20_–H_2_O case. These channels stand for the hot electron generation and intermolecular charge transfer pathways between the metal nanoparticle and water.

**FIGURE 5:**
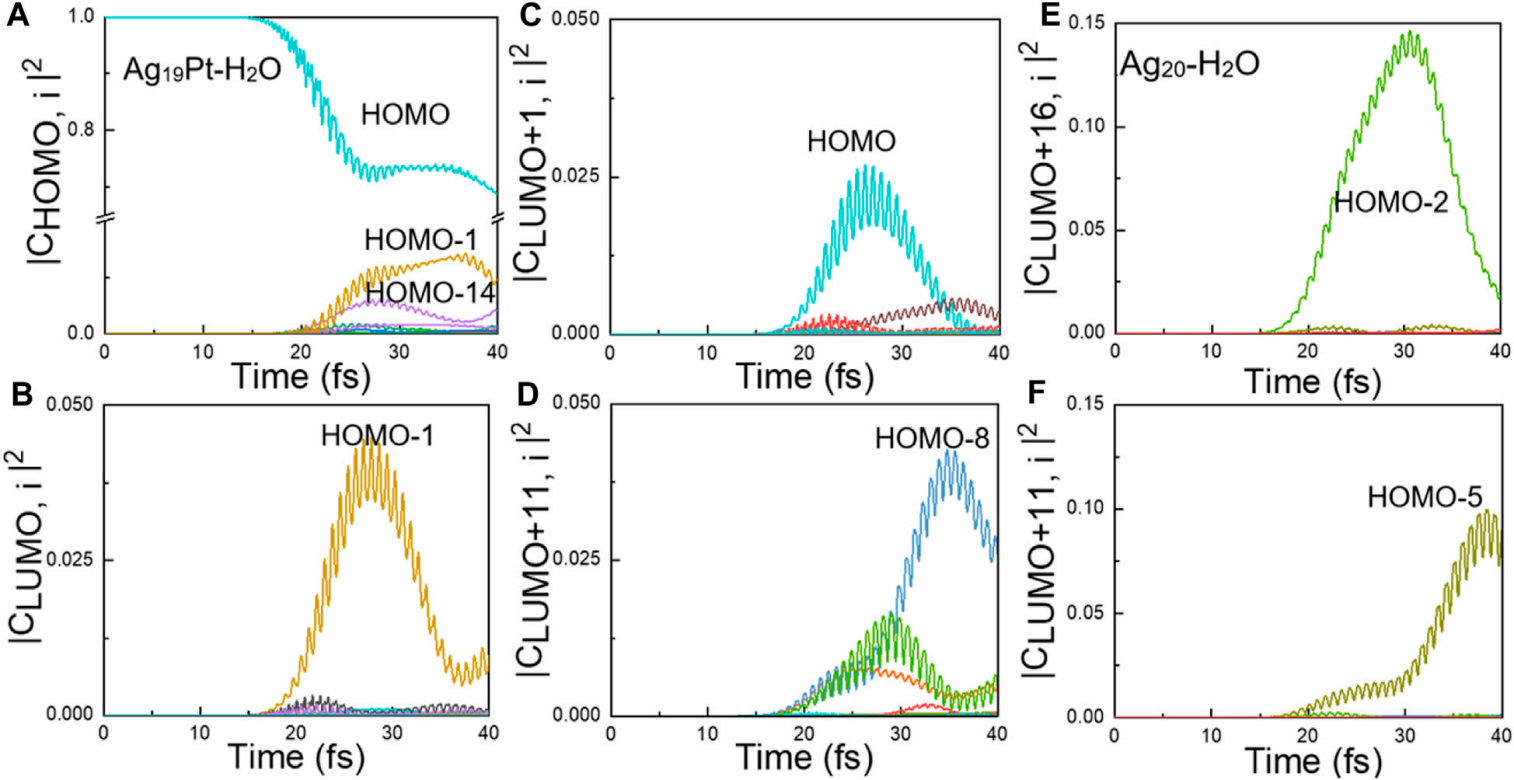
Ultrafast electron dynamics. Time-evolved transition coefficients from all the occupied states *i* to the Kohn–Sham state of **(A)** HOMO, **(B)** LUMO, **(C)** LUMO+1, and **(D)** LUMO+11 for Ag_19_Pt–H_2_O, respectively. The index *i* (*i* = 0–18) denotes the numerical order of the occupied states, while LUMO + *j* (*j* = 0–19) corresponds to the unoccupied states. Time-evolved transition coefficients from all the occupied states *i* to the state **(E)** LUMO+11 and **(F)** LUMO+16 for Ag_20_–H_2_O, respectively. The index *i* (*i* = 0–13) denotes the numerical order of the occupied states, while LUMO + *j* (*j* = 0–19) corresponds to the unoccupied states. Only the important orbitals that significantly contribute to the density change are labeled.

**Charge Transfer Mechanisms**. To confirm the underlying charge transfer mechanisms for the two systems, we calculated the time-evolved transition coefficients from all occupied states to the orbitals mentioned earlier, as shown in Figure 5. The charge transfer mechanism for the two systems is analyzed as follows.

**The Ag_19_Pt–H_2_O Case**. Given the fact that the total number of electrons in Ag_19_Pt–H_2_O is an odd number, the HOMO of the system is half-occupied in spin-unpolarized calculations. Figure 5A reveals that the main contribution to the HOMO level stems from the HOMO-1 level. In addition, the main wave function component of the two orbitals is dominantly originated from the metal nanoparticle, which can be deduced from Figure 4B. Similar results can be obtained by the same analysis, as shown in Figures 4B, 5B,C, that is, the HOMO-1 and HOMO levels make a dominant contribution to the excitation to the LUMO and LUMO+1 state, where the corresponding wave functions are mainly distributed on the metal nanoparticle. Therefore, the channels of indirect charge transfer can be open *via* inelastic electron tunneling (Christopher et al., 2011; Brongersma et al., 2015; Linic et al., 2015; Thrall et al., 2013; Mukherjee et al., 2013; Mukherjee et al., 2014; Zhang et al., 2018; Yan et al., 2015). Second, there is a significant charge transfer from the HOMO-8 to LUMO+11 states, as shown in Figure 5D. Through the analysis given in Figure 4B, the orbital component of HOMO-8 is mainly contributed by the metal cluster, while the LUMO+11 orbital is mainly distributed on the water molecule. Meanwhile, the energy gap of (LUMO+11)-(HOMO-8) is equal to the photoenergy corresponding to the highest absorption peak for Ag_19_Pt–H_2_O, implying that this is a direct charge transfer via localized surface plasmon resonances (Yan et al., 2011; Kale et al., 2013; Kale et al., 2014; Yan et al., 2016; Kumar et al., 2019). In summary, the introduction of the Pt atom opens up more channels of charge transfer including intramolecular, indirect, and direct charge transfer pathways, resulting in efficient charge transfer and subsequent water splitting.

**The Ag_20_–H_2_O Case**. Figures 5E,F show that there exists obvious charge transfer from the HOMO-5 and HOMO-2 states to the LUMO+11 and LUMO+16 states, respectively. In addition, the analysis of the wave function component, as shown in Figure 4D, shows that the HOMO-5 and HOMO-2 states are mainly located on the metal nanoparticle, while the LUMO+11 and LUMO+16 states are mainly located on the H_2_O molecule. Meanwhile, the energy gap between the HOMO-5, HOMO-2 and LUMO+11, LUMO+16 states equals to the photoenergy of the major peak in the photoabsorption spectra for the Ag_20_–H_2_O system. These facts suggest that the direct charge transfer is the main charge transfer mechanism for the Ag_20_–H_2_O case.

## Conclusion

In summary, we probe the difference in the underlying charge transfer mechanism for plasmon-driven water splitting for two representative systems: Ag_19_Pt–H_2_O and Ag_20_–H_2_O. Through the analysis of non-adiabatic molecular dynamic trajectories, we find that water may split within 40 fs after photoexcitation in the Ag_19_Pt–H_2_O case, where the O–H bond is only slightly elongated and does not break in the simulations for the Ag_20_–H_2_O system. Our ultrafast carrier dynamic analysis finds that there is more charge transferred to the H_2_O molecule in the Ag_19_Pt–H_2_O system than in the Ag_20_–H_2_O system. More importantly, it can be inferred that the introduction of single Pt atom in Ag_19_Pt–H_2_O strengthens the optical absorption as well as the interactions between the water and metal nanoparticle, opening up more charge transfer channels than that in Ag_20_–H_2_O, resulting in successful water splitting events. These results provide a new microscopic picture for solar water splitting and may facilitate the design of high-efficiency single-atom photocatalysts.

## Methods

### Numerical Calculations

Most of the calculations are carried out using the real-space TDDFT code OCTOPUS (Castro et al., 2006; Andrade et al., 2012; Andrade et al., 2015), with local density approximation (LDA) for the exchange correlation functional. The simulation zone is defined by assigning a sphere around each atom with a radius of 5.0 Å and a spacing of 0.25 Å between the grid points. Hartwigsen–Goedecker–Hutter pseudopotentials are used to represent the interactions between valence electrons and the atomic cores (Troullier and Martins, 1991). A time step of 0.002 fs is used in the calculations. An electromagnetic pulse (δ function) is used for the optical absorption spectrum. In the simulations, the H_2_O molecule is initially placed 3.0 Å away from the tip of Ag_19_Pt and Ag_20_, as shown in Figures 1A,B. The laser pulse is a Gaussian wave packet, *E* (*ω, t*) = *E* *_max_* exp $[-\frac{{(t-t_{0}}_{}{)}^{2}}{2\tau^{2}}$ cos (ωt − ωt_0_ + φ), where the phase *φ* = 0 and *τ* = 3.3 fs. The laser field reaches the maximum *E*_max_ at time *t*_0_ = 20 fs.

Ground-state DFT simulations were performed with the Vienna *Ab initio* Simulation Package (VASP) (Kresse and Furthmüller, 1996) to obtain ground-state properties, using a projector-augmented wave (PAW) pseudopotential in conjunction with the Perdew–Burke–Ernzerhof (PBE) functional (Perdew et al., 1996), and the plane-wave basis set with an energy cutoff at 400 eV. The atomic structure of two systems was positioned in a cubic supercell of 30 × 30 × 30 Å^3^ along three directions and fully relaxed until the force on each atom was less than 0.02 eV/Å. A Monkhorst–Pack k-point mesh of 1 × 1×1 was adopted for the calculations. There are other methods which can also deal with the excited state dynamics including plasmon excitations of metal nanoparticles and their couplings with molecules (Shao et al., 2006; Guan et al., 2018; Lian et al., 2018; You et al., 2021).

## Data Availability

The original contributions presented in the study are included in the article/Supplementary Material; further inquiries can be directed to the corresponding authors.
