# Supplementary material for: Plasmon-Induced Water Splitting on Ag-Alloyed Pt Single-Atom Catalysts
Source: Front Chem. 2021 Oct 25;9:742794. doi: 10.3389/fchem.2021.742794 (PMC8573343; doi:10.3389/fchem.2021.742794)
Supplement: Supplementary file 1 [file Presentation1.pdf]

Supplementary Information for

Plasmon-induced Water Splitting on Ag-Alloyed Pt Single-Atom Catalysts

Yimin Zhang[1,2], Daqiang Chen[2], Weite Meng[1,3], Shunfang Li[1*], Sheng Meng[2,4*]

[1] *Key Laboratory of Material Physics, Ministry of Education, School of Physics and Microelectronics, Zhengzhou University, Zhengzhou 450001, China*

[2] *Beijing National Laboratory for Condensed Matter Physics and Institute of Physics, Chinese Academy of Sciences, Beijing 100190, China*

[3] *School of Chemical Engineering, Anhui University of Science and Technology, Huainan 232000, Anhui, China*

[4] *School of Physical Sciences, University of Chinese Academy of Sciences, Beijing 100190, China*

*Correspondence authors. Email: sflizzu@zzu.edu.cn; smeng@iphy.ac.cn

The supplementary information includes:

Figures S1-S3

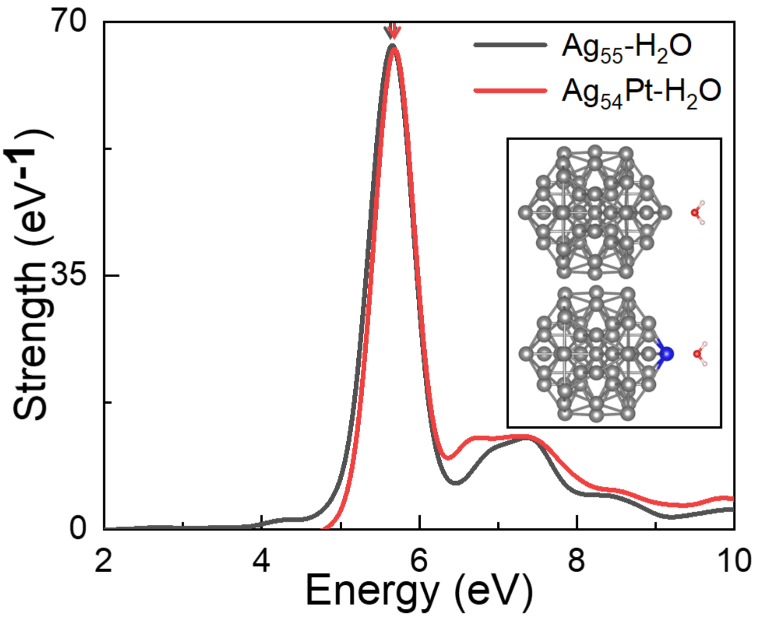

Figure S1. Absorption spectrum of Ag_{55}-H_2O, $Ag_{54}Pt$-H_2O, respectively. The inset denotes the atomic configuration of Ag_{55}-H_2O (up) and $Ag_{54}Pt$-H_2O (down).

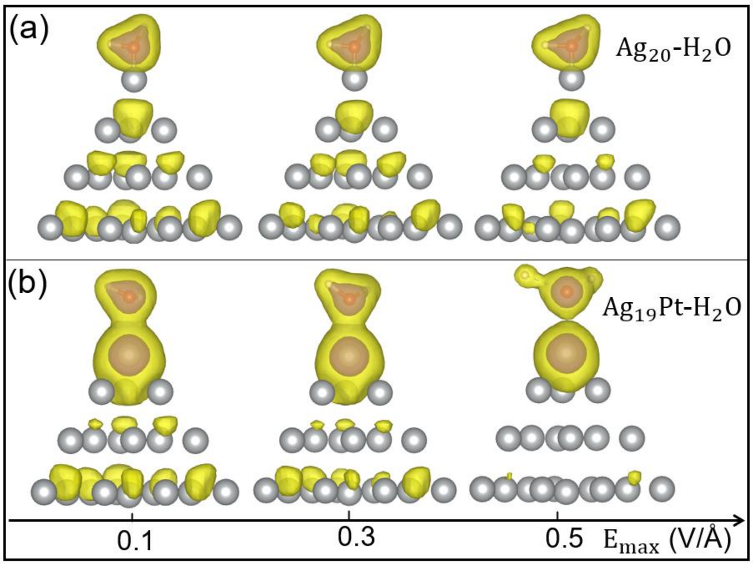

Figure S2. The charge density distribution for a) Ag_{20}-H_2O and b) $Ag_{19}Pt$-H_2O at t = 40 fs with different field strength of 0.1, 0.3 and 0.5 V/Å.

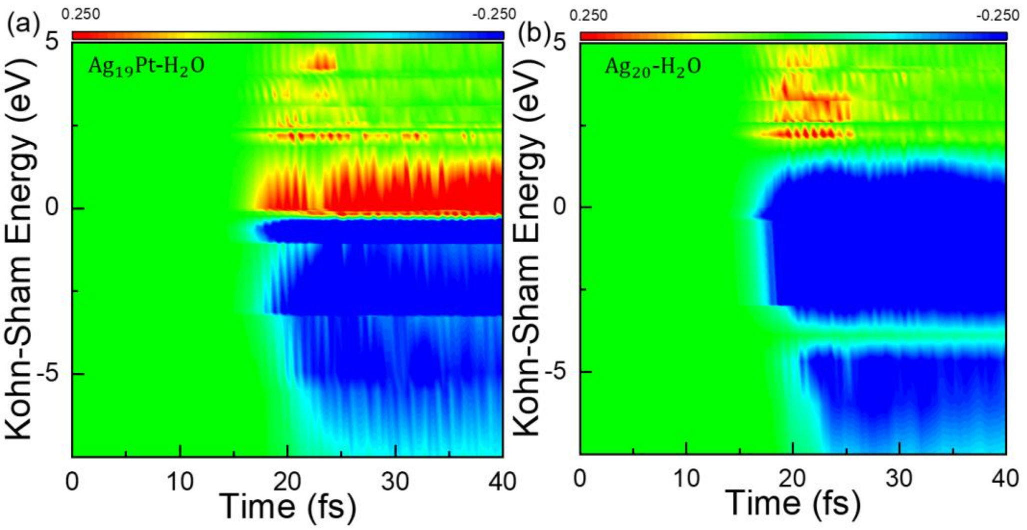

Figure S3. Time-dependent changes in the occupation of the KS states for a) $Ag_{19}Pt$-H_2O and b) Ag_{20}-H_2O with a field strength of 0.5 V/Å.
